# Prediction of S-Nitrosylation Modification Sites Based on Kernel Sparse Representation Classification and mRMR Algorithm

**DOI:** 10.1155/2014/438341

**Published:** 2014-08-12

**Authors:** Guohua Huang, Lin Lu, Kaiyan Feng, Jun Zhao, Yuchao Zhang, Yaochen Xu, Ning Zhang, Bi-Qing Li, Weiping Huang, Yu-Dong Cai

**Affiliations:** ^1^Institute of Systems Biology, Shanghai University, Shanghai 200444, China; ^2^Department of Mathematics, Shaoyang University, Shaoyang, Hunan 422000, China; ^3^School of Biomedical Engineering, Shanghai Jiaotong University, Shanghai 200240, China; ^4^Shanghai Center for Bioinformation Technology, Shanghai 200235, China; ^5^Graduate School of the Chinese Academy of Sciences, Beijing 100049, China; ^6^State Key Laboratory of Medical Genomics, Institute of Health Sciences, Shanghai Institutes for Biological Sciences, Chinese Academy of Sciences, Shanghai Jiao Tong University School of Medicine, Shanghai 200025, China; ^7^East China Normal University Software Engineering Institute, Shanghai 200062, China; ^8^Department of Biomedical Engineering, Tianjin University, Tianjin Key Lab of BME Measurement, Tianjin 300072, China; ^9^Key Laboratory of Systems Biology, Shanghai Institutes for Biological Sciences, Chinese Academy of Sciences, Shanghai 200031, China

## Abstract

Protein S-nitrosylation plays a very important role in a wide variety of cellular biological activities. Hitherto, accurate prediction of S-nitrosylation sites is still of great challenge. In this paper, we presented a framework to computationally predict S-nitrosylation sites based on kernel sparse representation classification and minimum Redundancy Maximum Relevance algorithm. As much as 666 features derived from five categories of amino acid properties and one protein structure feature are used for numerical representation of proteins. A total of 529 protein sequences collected from the open-access databases and published literatures are used to train and test our predictor. Computational results show that our predictor achieves Matthews' correlation coefficients of 0.1634 and 0.2919 for the training set and the testing set, respectively, which are better than those of k-nearest neighbor algorithm, random forest algorithm, and sparse representation classification algorithm. The experimental results also indicate that 134 optimal features can better represent the peptides of protein S-nitrosylation than the original 666 redundant features. Furthermore, we constructed an independent testing set of 113 protein sequences to evaluate the robustness of our predictor. Experimental result showed that our predictor also yielded good performance on the independent testing set with Matthews' correlation coefficients of 0.2239.

## 1. Introduction

Nitric oxide (NO) has been reported to be an important signaling molecule which involves physiological and pathophysiological regulations of some cellular processes, such as cardiovascular, respiratory, gastrointestinal, reproductive, and host defense [1–4]. Protein S-nitrosylation which is covalently modified by NO has recently been discovered to play important roles in regulating diverse pathways [5–7] and other biological activities [8], such as chromatin remodeling [9], transcriptional regulation [10], cellular trafficking [11], and apoptosis [12]. Also, it has been reported that aberrant S-nitrosylation might contribute to some diseases such as neurodegenerative disorders [1, 13] and cancers [14]. Several biochemical approaches have been developed to identify S-nitrosylation sites; for example, Forrester et al. [15] used RAC (resin-associated capture) method to isolate SNO protein, and Foster et al. [16] utilized an approach based on protein microarray to screen S-nitrosylation sites.

In contrast to time-consuming and labor-intensive experiments, computational approach is fast and cost-effective. It is reported that there have been at least 170 databases and computational tools concerned with posttranslational modification including protein S-nitrosylation modification [17]. With regard to predicting S-nitrosylation modification sites, Xue et al. [17] developed a software tool named GPS-SNO 1.0; Hao et al. [18] applied support vector machine (SVM), Lee et al. [19] used the maximal dependence decomposition- (MDD-) clustered SVMs, and Li et al. [20] utilized k-nearest neighbor algorithm to deal with the problem. Although computational approach is becoming more and more attractive, prediction of S-nitrosylation sites still remains a great challenge due to the complications of effectively protein encoding.

In the paper, we presented a new computational framework based on kernel sparse representation theory to predict S-nitrosylation sites. The framework consists of two steps: feature extraction and feature selection. Firstly, 666 features were extracted from five categories of amino acid properties, that is, sequence conservation, amino acid factor, secondary structure, solvent accessibility, and amino acid occurrence frequency, and one protein structure feature, the residual disorder. Then, a two-stage feature selection procedure was applied to select an optimal subset from the 666 redundant features. Finally, a webserver for the prediction of S-nitrosylation sites based on kernel sparse representation classification and minimum Redundancy Maximum Relevance algorithm is available at http://www.zhni.net/snopred/index.html.

## 2. Materials

The training and testing sets adopted in the paper were constructed as follows. A total of 645 protein sequences (see Supplementary Material S1 available online at http://dx.doi.org/10.1155/2014/438341) containing S-nitrosylation sites (see Supplementary Material S2) were first collected from open-access databases and the published literatures. Among the 645 protein sequences, 25 were from Uniprot database (version 2011_7) [21], 327 were from a research done by Xue et al. [17], and the other 293 protein sequences were from three recent reviews [22–24] on S-nitrosylation identification. The S-nitrosylation sites on the 645 protein sequences are all verified by experiments. Then, the sequence-clustering program CD-HIT [25] was applied to screen the 645 protein sequences. The cutoff value of CD-HIT was 0.4, meaning that the protein sequences having pairwise sequence identity greater than 40% to one another were removed. Finally, 529 protein sequences were left for analysis. Samples were then collected by taking peptides composed of 21 continuous residues with the central residue as cysteine; that is, peptides including a central cysteine and with each 10 residues in the upstream and downstream of the cysteine were picked out. For peptides with cysteine but which were less than 21 residuals, labels “X” were appended to end of the peptides. Thus, there were totally 2516 peptides obtained from the 529 proteins. 827 peptides with S-nitrosylation modification sites were labeled as positive samples and the remaining 1689 peptides were labeled as negative ones. More detailed information about collecting data can be found in our previous work [20]. The 2516 samples were grouped into training dataset and testing dataset at the ratio of 4 : 1; that is, we used 80% of the samples as the training samples, because sufficient samples were needed to train the predictor. Meanwhile, to evaluate the robustness, 20% of the samples were left for the testing. During sample grouping, positive samples and negative samples are distributed in a way so that the ratios of positive-to-negative samples in the training and testing datasets remained the same as that of the whole dataset which is about 1 : 2 (positive-to-negative ratio was 827 : 1689 in the whole date set). Consequently, the training set was composed of 662 positive and 1351 negative samples, and the testing set was composed of 165 positive and 338 negative samples (see Supplementary Materials S3 and S4).

Besides the training and testing sets mainly collected from published literatures, we also constructed an independent testing set with the Uniprot database of the latest version (version 2014_05). We searched the Uniprot database for those protein sequences with S-nitrosylation identification. Then, by deleting the proteins which had been used in the training and testing sets, totally 113 sequences containing S-nitrosylation sites were obtained. The 113 sequences were used as the independent testing set (see Supplementary Material S6). Thus, we could do comparison between different methods based on the independent testing set.

## 3. Methods

### 3.1. Feature Extraction

All features were derived from five categories of amino acid properties and one protein structure feature: (1) evolutionary conservation, (2) physicochemical or biochemical properties, (3) solvent accessibilities, (4) frequency around nitrosylated cysteine, (5) secondary structural properties, and (6) disorder status.

The evolutionary conservation of amino acid is very important, which is generally represented as the probability that it would mutate into other 20 kinds of amino acid. By using PSI-BLAST program [26], a 21 × 20 = 420 dimensional vector describing conservation of each peptide was obtained.

Physicochemical or biochemical properties of amino acid were characterized quantitatively as a 5-dimensional vector using amino acid index database [27], whose elements represent properties of polarity, secondary structure, molecular volume, codon diversity, and electrostatic charge, respectively. Except the cysteine, 20 amino acids in a peptide were represented as a 100-dimensional vector.

Disorder status of amino acid was quantified as a disorder score by the predictor of protein disorder [28], and thus, for a peptide, its disorder status was represented by a 21-dimensional vector.

Secondary structural properties, that is, “helix,” “strand,” and “others,” and the solvent accessibility, that is, “buried” and “exposed,” of an amino acid were calculated by the predicting software of protein structure and structural feature [29], resulting in a 5-dimensional encoding vector consisting of 0 or 1. A 21 × 5 = 105 dimensional vector represented the secondary structural and solvent accessibility properties of a peptide.

Frequency of the twenty amino acids around nitrosylated cysteine (nitrosylation site was excluded) was also taken into consideration.

Hence, each sample could be represented as a numerical vector containing as many as 666 (420 + 100 + 21 + 105 + 20) features.  Table 1 shows the distribution of features. Details of feature construction could be found in our previous work [20].

### 3.2. Feature Selection

A two-stage feature selection procedure is used to select optimal feature subset from the feature space. The predictor constructed by the optimal feature subset is our final S-nitrosylation sites predictor. The procedure is described as follows.

*Stage 1*. All features are evaluated by the minimum Redundancy Maximum Relevance (mRMR) algorithm [30] and then ranked according to their mRMR scores.

*Stage 2.* Based on the mRMR evaluation, incremental feature selection procedure [31, 32] is adopted to search for the optimal feature subset with the help of kernel sparse representation classification (KSRC) algorithm.

#### 3.2.1. mRMR Algorithm

The mRMR algorithm proposed by Peng et al. [30] is a feature evaluation method based on mutual information. Mutual information is able to quantify the dependency between two variables. The larger the mutual information is, the more the dependency between the two variables is. Mutual information between two random variables *X* and *Y* is defined as follows:

(1) MI(X,Y)=∬p(x,y)log⁡p(x,y)p(x)p(y)dx dy,

where function *p* denotes probabilistic or joint probabilistic density.

Mutual information between the feature space *Ω* = (*X*_1_, *X*_2_,…, *X*_*k*_) and the target variable *Y* is defined as follows:

(2) MI(Ω,Y)=∬p(Ω,y)log⁡p(Ω,y)p(Ω)p(y)ds dy=∫k+1⋯∬p(x1,x2,…,xk,y)    ×log⁡p(x1,x2,…,xk,y)p(x1,x2,…,xk)p(y)dx1 dx2⋯dxk dy.

The mRMR algorithm aims to evaluate feature subsets *S* and then selects the optimal feature subset that meets the minimal redundancy and maximal relevance criteria, that is, the minimal dependency to the entire feature space and the maximal dependency to the target variable *Y*. Minimal redundancy to the entire feature space can be calculated by the following equation:

(3) $$\begin{array}{c} \underset{S\subseteq \Omega }{\min}\frac{1}{{\left|S\right|}^{2}}\sum_{X_{i},X_{j}\in S}\text{MI}\left(X_{j},X_{i}\right). \end{array}$$

Maximal dependency to the target variable *Y* can be calculated by the following equation:

(4) $$\begin{array}{c} \underset{S\subseteq \Omega }{\max}\frac{1}{\left|S\right|}\underset{X_{j}\in S}{\sum }\text{MI}\left(X_{j},Y\right). \end{array}$$

Thus, the mRMR evaluation can be quantified as score by integrating (3) and (4) into the following equation:

(5) $$\begin{array}{c} \underset{S\subseteq \Omega }{\max}\left\{\frac{1}{\left|S\right|}\sum_{X_{j}\in S}\text{MI}\left(X_{j},Y\right)-\frac{1}{{\left|S\right|}^{2}}\sum_{X_{i},X_{j}\in S}\text{MI}\left(X_{j},X_{i}\right)\right\}. \end{array}$$

#### 3.2.2. Incremental Feature Selection

In the implementation, the mRMR criterion is hard to satisfy, especially when the feature space is large. Hence, to attain an optimal feature subset of minimal redundancy and maximal relevance, a heuristic strategy named incremental feature selection [31, 32] is adopted for the search of feature subset.

Firstly, all the features are scored by (5), by shrinking feature subset *S* to contain only one feature. Secondly, arrange all the features according to their mRMR scores. Thirdly, search for optimal feature subset by an increment means as follows.

Suppose all the features in the feature space *Ω* have been arranged in the order from high mRMR score to low mRMR score. Beginning from the feature of the highest mRMR score, move features from the scored feature space to the selected feature subset sequentially. When one feature is added, evaluate the classification performance of the feature subset by predictors which are constructed by the KSRC algorithm (see Section 3.2.4 for details). Finally, the feature subset of the highest classification performance is selected as the optimal feature subset and the predictor constructed by the optimal feature subset is the final predictor. In this study, the method used to evaluate the classification performance is presented in Section 3.2.3.

#### 3.2.3. Evaluation Metrics

Four indicators, sensitivity (SN), specificity (SP), accuracy (ACC), and Matthews' correlation coefficient (MCC), are used to evaluate the performance of predictors when new features are added. Consider the following:

(6) SN=TP(TP+FN),SP=TN(TN+FP),ACC=(TP+TN)(TP+FN+FP+TN),MCC=(TP×TN−FN×FP)(TP+FN)(TP+FP)(TN+FP)(TN+FN).

TP and TN represent the numbers of true positive and true negative, respectively. FP and FN represent the numbers of false positive and false negative, respectively. Among the four indicators, MCC is the most significant indicator, which is used to optimize the procedure of feature selection in this study.

#### 3.2.4. KSRC Algorithm

In this paper, KSRC algorithm is applied to construct predictor. The KSRC algorithm integrates the sparse representation classification (SRC) algorithm and the kernel function technique to fulfill classification task [33, 34]. In the following section, we will introduce the SRC algorithm and the kernel function technique, respectively, and then illustrate how to integrate the two techniques.

In the recent years, the SRC algorithm has been successfully applied in these fields of signal recovery, signal encoding, and signal classification [33–41]. The principle underlying the SRC algorithm is that testing samples can be represented as linear combination of training samples if the testing and training samples belong to the same category so that the representation coefficient of a testing sample under all training samples might supply sufficient information to determine the category of the testing samples.

Suppose there are *c* distinct classes, each with *n*_*k*_ samples, *k* = 1,2,…, *c*. And *X* ^*k*^ = (*x*_1_^*k*^, *x*_2_^*k*^,…, *x*_*n*_*k*__^*k*^) is a matrix consisting of samples from the *k*th class, where *x*_*j*_^*k*^ (1 ≤ *j* ≤ *n*_*k*_) is a column vector, representing the *j*th sample in the class *k*. All training samples are concatenated to form a matrix *X* = [*X* ^1^, *X* ^2^,…, *X* ^*c*^]. Computing the sparsest coefficient vector *α* of a test sample *y* under the matrix *X* is modeled as follows:

(7) $$\begin{array}{c} \min{||\alpha ||}_{0},\;\text{subject}\;\text{to}\;y=X\alpha \end{array}$$

or

(8) $$\begin{array}{c} \min{||\alpha ||}_{0},\;\text{subject}\;\text{to}\;{||y-X\alpha ||}_{2}\leq \varepsilon , \end{array}$$

where operator ||•||_0_ denotes the *l*_0_ norm, which counts nonzero entries, and operator ||•||_2_ denotes the *l*_2_ norm of a vector, respectively.

Since the pursuit of exact solution of (7) and (8) is an NP-hard problem [42], the orthogonal matching pursuit (OMP) [43, 44] algorithm is used to seek an approximate solution to (7) and (8) in our works. The OMP is an iterative greedy method. Each step of iteration in OMP algorithm contains three operations: (1) computing residual referring to difference between original signal and recovery one, (2) selecting the column with the highest correlation to the current residual, and (3) projecting original signal into the linear subspace spanned by these already selected columns. For convenient description, the following symbols were used. The symbol *X* specified a matrix, *X*_*t*_ referred to the column *t* in the matrix, and *X*_Θ_ consisted of columns of the matrix *X* with the indices Θ. The OMP algorithm is described in Algorithm 1.

Once a coefficient vector *α* was gained by the OMP algorithm, the category of the corresponding testing sample was determined by the following rule:

(9) $$\begin{array}{c} K=\underset{k=1,2,\ldots ,c}{\arg\;\min}{||y-X\alpha_{k}||}_{2}, \end{array}$$

where *α*_*k*_ = (0,0,…, 0, *α*_1_^*k*^, *α*_2_^*k*^,…, *α*_*n*_*k*__^*k*^,…, 0) was a coefficient whose entries were all zero except *α*_*i*_^*k*^ (1 ≤ *i* ≤ *n*_*k*_) which corresponds to the samples from the class *k* and is equal to the corresponding element from *α*. The details of the SRC algorithm were shown in Algorithm 2.

Nevertheless, the performance of the SRC algorithm might be limited, if the testing samples are not linearly representable in the space of training sample [34]. Therefore, in our work, kernel function technique is applied to project testing sample into higher-dimensional space so as to alter the distributed structures of the samples.

Kernel function technique is a widely used technique that is able to map data from low-dimensional space to higher-dimensional space [34]. A well-chosen kernel function enables original linearly inseparable samples to become linearly separable in the high-dimensional feature space. In our work, the Laplacian kernel function Ψ(*x*, *y*) = *e* ^−|*x*−*y*|/*δ*^ was employed.

Assume that the training samples with *c* classes *X* = [*X* ^1^, *X* ^2^,…, *X* ^*c*^] = [*x*_1_, *x*_2_,…, *x*_*n*_] as previously shown and the testing sample *y* are mapped to high-dimensional data Ψ(*X*) = [Ψ(*X* ^1^), Ψ(*X* ^2^),…, Ψ(*X* ^*c*^)] = [Ψ(*x*_1_), Ψ(*x*_2_),…, Ψ(*x*_*n*_)] and Ψ(*y*), respectively. Similar to (7), the problem with the sparest coefficient representation of Ψ(*y*) under Ψ(*X*) was formulated as follows:

(10) $$\begin{array}{c} \min{||\alpha ||}_{0},\;\text{subject}\;\text{to}\;\Psi \left(y\right)=\Psi \left(X\right)\alpha . \end{array}$$

Let Π = [Ψ(*x*_1_), Ψ(*x*_2_),…, Ψ(*x*_*n*_)]^*T*^ be a column vector. Equation Ψ(*y*) = Ψ(*X*)*α* left multiplied by Π was rewritten as

(11) $$\begin{array}{c} \left[\begin{array}{c} \Psi \left(y\right)\Psi \left(x_{1}\right)\\ \vdots \\ \Psi \left(y\right)\Psi \left(x_{n}\right) \end{array}\right]=\left[\begin{array}{ccc} \Psi \left(x_{1}\right)\Psi \left(x_{1}\right) & \cdots & \Psi \left(x_{1}\right)\Psi \left(x_{n}\right)\\ \vdots & \cdots & \vdots \\ \Psi \left(x_{n}\right)\Psi \left(x_{1}\right) & \cdots & \Psi \left(x_{n}\right)\Psi \left(x_{n}\right) \end{array}\right]\alpha . \end{array}$$

According to the properties of kernel function, (11) is further expressed as

(12) $$\begin{array}{c} \left[\begin{array}{c} \Psi \left(y,x_{1}\right)\\ \vdots \\ \Psi \left(y,x_{n}\right) \end{array}\right]=\left[\begin{array}{ccc} \Psi \left(x_{1},x_{1}\right) & \cdots & \Psi \left(x_{1},x_{n}\right)\\ \vdots & \cdots & \vdots \\ \Psi \left(x_{n},x_{1}\right) & \cdots & \Psi \left(x_{n},x_{n}\right) \end{array}\right]\alpha . \end{array}$$

Therefore, minimum equation (10) is equivalent to

(13) $$\begin{array}{c} \min{||\alpha ||}_{0},\;\text{subject}\;\text{to}\;\text{(8).} \end{array}$$

Equation (13) has the same solution as (10). The KSRC was shown in Algorithm 3.

## 4. Results and Discussion

### 4.1. Optimal Feature Subset Selection

First, the mRMR algorithm [30] was applied to the training set, producing a sequence of 666 scored features. Details of the results can be found in Supplementary Material S5.

**Figure 1:**
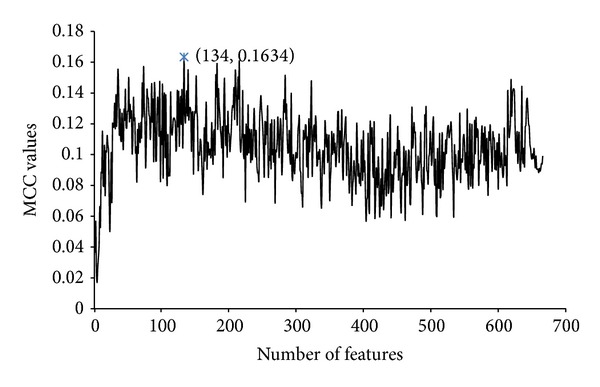
MCC value of 10-fold cross validation of the KSRC on the training set in the incremental feature selection procedure.

Second, apply incremental feature selection procedure to search optimal feature subset.  Figure 1 shows MCC values of each candidate feature subset by using 10-fold cross validation on the training set. The best MCC value is 0.1634, corresponding to the combination of the first 134 features. Therefore, this candidate feature subset was regarded as the optimal subset.

In the implementation, the factor *δ* of the Laplacian kernel function in the KSRC algorithm is 100. The sparsity *k* in OMP algorithm was 50. The used OMP algorithm codes are available at the following site: http://www.cs.technion.ac.il/~ronrubin/software.html [45]. The used mRMR codes are available at http://penglab.janelia.org/proj/mRMR/ [30].

### 4.2. Comparison with Other Algorithms

As was mentioned in Section 1, quite a few methods have been developed to predict the S-nitrosylation sites in recent years. However, it was difficult to make direct comparisons between them due to the following two reasons. First, different methods usually employed different datasets. It was biased to compare their overall performances based on different datasets. Secondly, we did not know what parameters they used to optimize the predictors. So, it was difficult for us to compare other methods with ours based on the same training and testing datasets.

Notwithstanding this, we attempted to compare our methods with other data mining methods based on our training and testing datasets. Hence, the KSRC algorithm proposed in this paper was compared to five other data mining algorithms: SRC [38], k-nearest neighbor algorithm (KNN) [46], random forest (RF) [47], sequential minimal optimization (SMO) [48], and Dagging [49]. KNN is an instance-based learning algorithm, which is widely used due to its simplicity and efficiency in training. RF is an integration method by combining many tree predictors together. Each tree predictor performs computation based on the values of a random vector sampled independently and with the same distribution for all trees in the forest. SMO is an algorithm that trains the support vector machine. Dagging is an algorithm that ensembles weak classifiers. In terms of implementation, KSRC and SRC were coded in Matlab language by virtue of the OMP package [45]. The computation of KNN, RF, SMO, and Dagging algorithms was performed by Weka (version 3-6-1) [50], which is a collection of learning machine algorithms and is available at http://www.cs.waikato.ac.nz/ml/weka/. In this work, the number of the nearest neighbors in the KNN is 3. The RF, SMO, and Dagging use the default parameters in the Weka. The sparsity of the OMP in the SRC is 50, the same as that of the KSRC. All the computer programs were executed on the Operation System platform Fedora 17.

**Figure 2:**
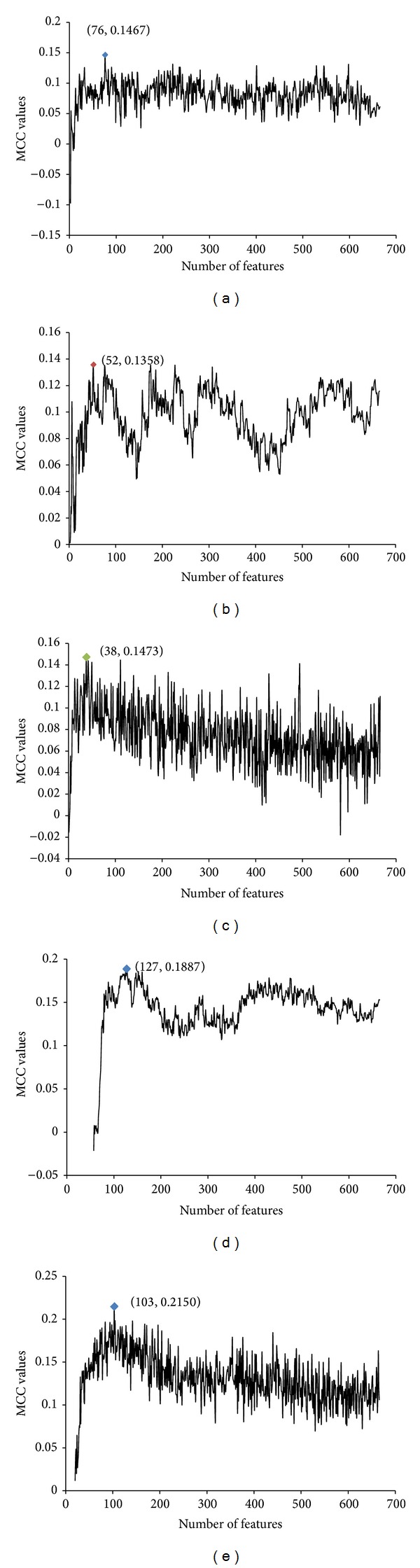
MCC curves of 10-fold cross validation on the training set of (a) SRC, (b) KNN, (c) RF, (d) SMO, and (e) Dagging in the incremental feature selection procedure.

The four indicators, SN, SP, ACC, and MCC, mentioned in Section 3.2.3, were also used for the comparison of different algorithms. The MCC curves of SRC, KNN, RF, SMO, and Dagging on the training set were plotted in Figure 2. The five algorithms attained optimal feature subsets containing 76, 52, 38, 127, and 103 features, respectively. All six algorithms were compared both on the training set and on the testing set with optimal feature subsets of their own. Tables 2 and 3 showed their performances on the training and testing datasets, respectively. As indicated by Table 2 and Figure 2, KSRC could achieve MCC that exceeded 0.16 on the training set. Although SMO and Dagging performed better in terms of the MCC, KSRC showed better SN than that of SMO and Dagging.  Table 3 presented the performances of the six algorithms on the testing dataset, which were not previously used in the training. As shown in Table 3, KSRC yielded the highest MCC and SN among all of the six algorithms, while SMO and Dagging showed poor MCC on the testing set. The high MCC and SN of KSRC on both the training and testing datasets indicated that KSRC was more effective and robust than the other five data mining algorithms.

To compare the predictive performances of the 134 optimal features with that of the original 666 features, the 10-fold cross validation and independent tests were also conducted on the training and testing sets by the 666 original features, respectively.  Table 4 shows the performance of using original 666 features on the training and testing sets, respectively. It can be seen in Table 4 that SN and MCC with the 134 optimal features were much better than those of the original features, though SP is a bit worse. Since the MCC is the most important criterion among the adopted metrics, we conclude that the 134 optimal features performed better than the original 666 features.

### 4.3. Comparison of Algorithms on Independent Testing Set

Since the training and testing sets were mainly collected from published literatures, we constructed an independent testing set for the comparison between our method and other methods. The independent testing set contained 113 protein sequences from the latest version of Uniprot database (version 2014_05) (see Section 2 for details). Two existing S-nitrosylation predictors, iSNO-AAPair [51] and iSNO-PseAAC [52], were used for comparison. The comparison results of our predictor, iSNO-AAPair, iSNO-PseAAC, and other five data mining algorithms on the independent testing set were presented in Table 5. As shown in Table 5, the SRC algorithm achieved the highest MCC of 0.2617, and our proposed KSRC algorithm was the second with MCC of 0.2239. The iSNO-AAPair and iSNO-PseAAC predictors attained MCC of 0.1125 and 0.1190, respectively, both of which were only approximately half of the KSRC algorithm. Although the MCC of KSRC algorithm was a little lower than that of SRC algorithm, the KSRC algorithm was the one algorithm that could achieve high and stable performance in both of the testing set and the independent set (as shown in Tables 3 and 5), demonstrating the robustness of the KSRC algorithm among different datasets.

## 5. Conclusions

In the paper, we proposed a framework based on the KSRC to computationally identify S-nitrosylation modification sites. Our experimental results show that KSRC outperforms other state-of-the-art algorithms in terms of the key prediction metrics. The KSRC is an application of kernel function technique to the SRC. Kernel approach can project linearly inseparable samples into high-dimensional feature space with the use of kernel functions. If an appropriate kernel function is selected, the original linearly inseparable samples could become linearly separable in the high-dimensional feature space. Kernelizing of the sparse representation by Laplacian function could improve the separability of the samples and yields higher MCC than those linear classification algorithms, such as KNN and SRC. We believe that the proposed KSRC based framework could become a helpful tool for the prediction and analyses of protein S-nitrosylation.

## Supplementary Material

supplementary material S1:lists 645 protein sequences containing S-nitrosylation sites.
supplementary material S2:lists both of the protein sequence Uniprot ids and S-nitrosylation site locations in the training and testing sets, respectively.
supplementary material S3:presents the training set with 666 features.
supplementary material S4:presents the testing set with 666 features.
supplementary material S5:lists the order of the 666 features ranked by the mRMR algorithm.

## Figures and Tables

**Algorithm 1:**
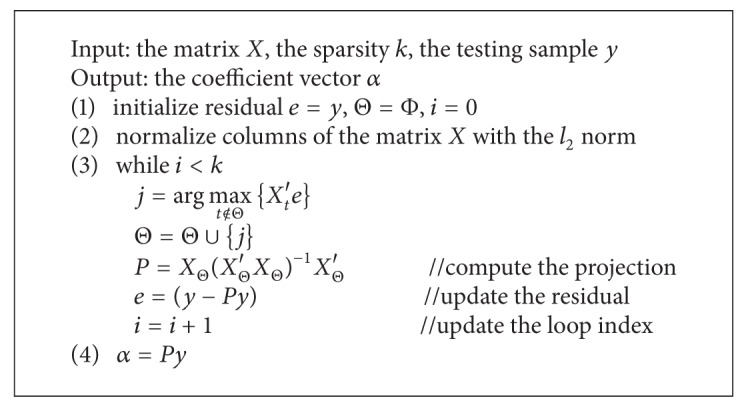
OMP algorithm.

**Algorithm 2:**
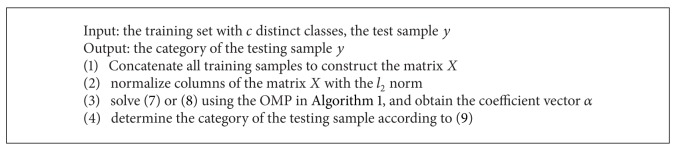
SRC algorithm.

**Algorithm 3:**
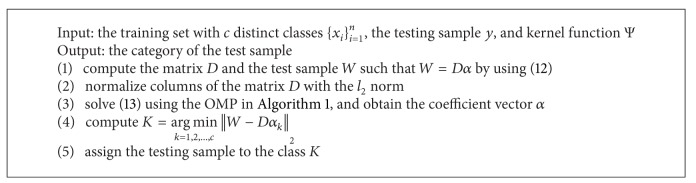
KSRC algorithm.

**Table 1:** Distribution of feature type for a sample.

Feature category	Number of features from each category
Evolutionary conservation	21 × 20
Amino acid factor	20 × 5
Secondary structure	21 × 3
Solvent accessibility	21 × 2
Amino acid frequency	20 × 1
Disorder	21 × 1
Number of features of a sample	666

**Table 2:** Performances of six algorithms on the training set with the respective optimal features using 10-fold cross validation.

	SN	SP	ACC	MCC
KSRC	0.4048	0.7543	0.6393	0.1634
SRC	0.3489	0.7876	0.6433	0.1467
KNN	0.3852	0.7469	0.6279	0.1358
RF	0.3399	0.7957	0.6458	0.1473
SMO	0.2840	0.8705	0.6776	0.1887
Dagging	0.3610	0.8320	0.6771	0.2150

KSRC: kernel sparse representation classification; SRC: sparse representation classification; KNN: *k*-nearest neighbor algorithm; RF: random forest method; SMO: sequential minimal optimization; Dagging refers to the use of majority vote to combine multiple models derived from a single learning algorithm using disjoint samples.

**Table 3:** Performances of six algorithms on the testing set with the respective optimal features.

	SN	SP	ACC	MCC
KSRC	0.4727	0.8077	0.6978	0.2919
SRC	0.2909	0.7988	0.6322	0.1000
KNN	0.4061	0.7899	0.6649	0.2062
RF	0.3636	0.8343	0.6799	0.2206
SMO	0.2364	0.8669	0.6600	0.1299
Dagging	0.2848	0.8343	0.6541	0.1386

**Table 4:** Performances of KSRC on the training and testing sets with the original 666 features.

	SN	SP	ACC	MCC
The training set	0.2749	0.8120	0.6354	0.0991
The testing set	0.2909	0.8462	0.6640	0.1612

**Table 5:** Performances of eight algorithms on the independent testing set with the respective optimal features.

	SN	SP	ACC	MCC
KSRC	0.5196	0.7368	0.6915	0.2239
SRC	0.5588	0.7419	0.7038	0.2617
KNN	0.4069	0.7419	0.6721	0.1333
RF	0.4657	0.7535	0.6936	0.1958
SMO	0.1765	0.8645	0.7211	0.0474
Dagging	0.2745	0.7884	0.6813	0.0612
iSNO-AAPair	0.4020	0.7252	0.6578	0.1125
iSNO-PseAAC	0.5343	0.6103	0.5945	0.1190
